# Digestibility of gluten proteins is reduced by baking and enhanced by starch digestion

**DOI:** 10.1002/mnfr.201500262

**Published:** 2015-08-21

**Authors:** Frances Smith, Xiaoyan Pan, Vincent Bellido, Geraldine A. Toole, Fred K. Gates, Martin S. J. Wickham, Peter R. Shewry, Serafim Bakalis, Philip Padfield, E. N. Clare Mills

**Affiliations:** ^1^ Centre for Respiratory Medicine and AllergyInstitute of Inflammation and RepairManchester Institute of BiotechnologyUniversity of ManchesterManchesterUK; ^2^ Institut Polytechnique laSalle BeauvaisBeauvaisFrance; ^3^ Institute of Food ResearchNorwichUK; ^4^ Campden BRIChipping CampdenUK; ^5^ Reacta Biotech LimitedManchesterUK; ^6^ Rothamsted ResearchHarpendenUK; ^7^ School of Chemical EngineeringUniversity of BirminghamBirminghamUK

**Keywords:** Allergen, Baking, Celiac, Digestion, Gluten

## Abstract

**Scope:**

Resistance of proteins to gastrointestinal digestion may play a role in determining immune‐mediated adverse reactions to foods. However, digestion studies have largely been restricted to purified proteins and the impact of food processing and food matrices on protein digestibility is poorly understood.

**Methods and results:**

Digestibility of a total gliadin fraction (TGF), flour (cv Hereward), and bread was assessed using in vitro batch digestion with simulated oral, gastric, and duodenal phases. Protein digestion was monitored by SDS‐PAGE and immunoblotting using monoclonal antibodies specific for celiac‐toxic sequences (QQSF, QPFP) and starch digestion by measuring undigested starch. Whereas the TGF was rapidly digested during the gastric phase the gluten proteins in bread were virtually undigested and digested rapidly during the duodenal phase only if amylase was included. Duodenal starch digestion was also slower in the absence of duodenal proteases.

**Conclusion:**

The baking process reduces the digestibility of wheat gluten proteins, including those containing sequences active in celiac disease. Starch digestion affects the extent of protein digestion, probably because of gluten‐starch complex formation during baking. Digestion studies using purified protein fractions alone are therefore not predictive of digestion in complex food matrices.

**Abbreviations**
BCIP: 5‐bromo‐4‐chloro‐3’‐indolylphosphate p‐toluidine salt
CD: celiac disease
DU: duodenal undigested control
GU: gastric undigested control
HMW: high molecular weight
HSA: human salivary amylase
LDS: lithium dodecyl sulfate
LMW: low molecular weight
MeOH: methanol
NBT: nitro‐blue tetrazolium chloride
SGF: simulated gastric fluid
SSF: simulated salivary fluid
TGF: total gliadin fraction

## 1 Introduction

Wheat is one of the most widely consumed cereals in the world and is a primary source of macronutrients due to its carbohydrate (in the form of starch) and protein content. Around half the grain protein comprises gluten, a protein fraction insoluble in water and 0.5M NaCl 1 that is now know to correspond to the prolamin and glutelin fractions first described by Osborne in his classification of plant proteins 2. Gluten proteins are classically divided into two groups, termed gliadins (prolamins) and glutenins (glutelins) and comprise‐related proteins that can be classified as prolamins based on their high contents of glutamine and proline and their solubility, in alcohol‐water mixtures following reduction 3. The monomeric gliadins comprise the ω‐, α‐, and γ‐ gliadins based on their mobility on electrophoresis at low pH while the polymeric types include high molecular weight (HMW) and low molecular weight (LMW) subunits of glutenins that are linked by intermolecular disulphide bonds. The LMW subunits, α‐gliadins and γ‐gliadins have related amino acid sequences and are termed S‐rich prolamins while the ω‐gliadins contain little cysteine and methionine and are termed S‐poor prolamins. All wheat gluten proteins contain extensive repeating sequences based on proline and glutamine rich motifs, with additional sequence similarity between the N‐ and C‐terminal domains of the S‐rich prolamins and HMW subunits of glutenin that share the common cysteine skeleton of the prolamin superfamily.

Wheat‐based foods can trigger immune‐mediated adverse reactions including both IgE‐mediated food allergies and the gluten intolerance syndrome celiac disease (CD). Thus, nonspecific lipid transfer proteins, α‐amylase inhibitors, gliadins (notably ω‐5 gliadin), LMW, and HMW subunits of glutenin have all been implicated as triggers of IgE‐mediated food allergy 4. In addition, the gluten proteins play an important role in eliciting CD 5, 10, a T‐cell mediated condition that is triggered by the presence of numerous T‐cell reactive epitopes found in the repetitive domain of prolamins 6, 7. Resistance to digestion may play a role in determining the ability of proteins to act as allergens, including their capacity to sensitize individuals in IgE‐mediated allergies 8, 9 and initiate the complex autoimmune responses underlying CD 5, 10. This characteristic is shared by many food allergens 11, 12, 13, although exceptions have been described 14. For example, in vitro digestion of recombinant α‐2 gliadin results in the formation of a stable 33 residue polypeptide containing three distinct T‐cell epitopes important for celiac disease 15, while the wheat LTP allergen, Tri a 14, is highly resistant to digestion 16.

Food processing may modify the susceptibility of a protein to digestion directly, by causing protein unfolding and aggregation, as well as modulating it as a consequence of interactions with other food components that form the food matrix 17, 18. For example, starch digestibility may be modified by the presence of proteins in foods 19, 20, 21, 22. Despite their importance in triggering immune‐mediated adverse reactions to foods, few studies have been published on the simulated gastro‐intestinal digestion of cereal foods and include raw and heated flour matrices 23, as well bread dough, crumb, and crust 24. Data suggest that baking modifies the immunological and physiochemical properties of cereal proteins, which seem to be less readily digested after baking, but variation between digestion protocols makes comparisons between studies difficult.

Traditionally in vitro digestion models mimicking the processes that take place in the oral, gastric, and duodenal compartments have taken the form of batch, test tube‐based models. The composition of the simulated digestive fluid can affect the rates of digestion, including pH, protease: protein ratios and inclusion of biosurfactants, such as phospholipid vesicles and bile salts. For example inclusion of phospholipid vesicles can increase the resistance of purified proteins to digestion in vitro 25, 26. Recently, other more physiologically relevant dynamic models have been built using echo planar imaging or computation model assistance including the Dynamic Gastric Model and Small Intestinal Model, respectively 27, 28. In an initial study, we have used a batch digestion model, adapted from one designed to investigate the digestion of purified proteins, to define the effect of baking on the digestibility of wheat gluten proteins. mAbs specific for the epitopes QQSF and QPFP, which are present in the CD toxic peptides from gliadins and LMW subunits of glutenin, were used to monitor digestibility of CD toxic gluten polypeptides. Subsequently, this model system was used to investigate how gluten digestibility may be modulated by digestion of starch.

## 2 Materials and methods

### 2.1 Materials

Enzymes used for digestion studies and their activities as provided by the manufacturer are listed in Supporting Information Table 1.

Twelve percent of NuPAGE Bis‐tris gels, NuPAGE lithium dodecyl sulphate (LDS) buffer (4X, pH 8.4), and SimplyBlue^TM^ safestain were from Invitrogen (Shropshire, UK). Mark 12^TM^ marker and SeeBlue^TM^ prestained marker were also from Invitrogen (Supporting Information Table 2). Secondary anti‐mouse IgG labeled with alkaline phosphatase and nitro‐blue tetrazolium chloride (NBT)/5‐bromo‐4‐chloro‐3' indolylphosphate p‐toluidine salt (BCIP) substrate solution were sourced from ThermoScientific (Leicestershire, UK). Egg lecithin (90%) was also sourced from Fisher Scientific (Leicestershire, UK) and used to prepare vesicles using sonication in simulated gastric fluid (SGF; Bandelin SONOPLUS HD 3200 with TT13 probe from Sigma‐Aldrich (Dorset, UK) as described previously 25. Purified total gliadin fraction was prepared as previously described 29 from wheat flour (*T. aestivum*) cv. Hereward and solubilized in DMSO prior to digestion. Grain (cv. Hereward) was supplied by Rothamsted Research (Hertfordshire, UK) and milled by Campden BRI (Gloucestershire, UK) to 81.4% extraction to provide white flour. This was used to prepare bread (100 g flour, 2 g yeast, 1.5 g salt, 1 g Bako fat emulsion, 0.01 g ascorbic acid) with fungal α‐amylase (Bakezyme P180. DSM, Delft, Netherlands) added to 80 Farrand Units (based on Hagberg Falling Number) and water added to the Brabender Farinograph (600 line) water absorption value using the Chorleywood bread process.

Monoclonal antibodies (mAbs) IFRN 0065 and 0610 (recognizing epitopes QQSF and QPFP, respectively, specific to gliadins and LMW glutenins) were used as culture supernatants 30.

### 2.2 Batch in vitro digestion

Digestions were performed using an in vitro batch digestion model that encompassed three compartments based on previously described models 26. Oral/gastric digestion was performed in duplicate and oral/gastro‐duodenal digestion in triplicate.

*Model chew*: Simulated salivary fluid (SSF, 0.15 M NaCl containing 6 μg/mL lysozyme, human salivary amylase (HSA) 29.7 U/g flour/bread carbohydrate, 3 mM urea, pH 6.9) was warmed to 37°C and added to flour/bread to mimic chewing. Specifically flour (2.08 g) was mixed to a paste with 3.87 mL water and 1.23 mL SSF containing 46.3 U HSA to form a “chew.” Frozen bread was thawed and cut into approximately 4 cm^3^ pieces without crusts and 35 g added to 12.25 mL of SSF containing 436 U HSA before mincing (Eddingtons mincer pro, product 86002, Berkshire, UK) for 30 s. Subsequently, 24.5 mL of deionized water was added to the minced bread and mixed by hand for an additional 1 min to simulate chewing. Samples of the “chew” were taken for starch and protein analysis, with protein samples chilled on ice for 10 min prior to freezing at –20°C until required for analysis. The remaining “chew” was then aliquoted (flour –7.18 g; bread –6.56 g) on ice prior to gastric digestion. Separate oral in vitro digests were performed in triplicate in the absence of HSA.

*Gastric digestion*: Simulated gastric fluid (SGF, 0.9 mM NaH_2_PO_4_, 3 mM CaCl_2_, 0.1 M HCl, 0.15 M NaCl, 16 mM KCl, pH 2.5) was prepared alone (GU, gastric undigested control) or containing either 182 U (highP SGF) or 63 U (lowP SGF) of pepsin per mg of gliadin/flour/bread protein to be digested. Prewarmed (37°C) SGF alone, highP SGF or lowP SGF was then added (1900 μL) to 100 μL aliquots of purified total gliadins (20 mg/mL in DMSO). Samples were placed in a shaking incubator at 37°C, 170 rpm and incubated for 0.3 min (all samples), 11, 22, 33, 44, 55, 66, 77, and 120 min (only pepsin‐containing incubations). Digestion was stopped by raising the pH to 7.5 by addition of 0.5 M NaHCO_3_. For “chewed” flour or bread respectively 3.37 mL or 3 mL of SGF alone, highP SGF or lowP SGF was added to each aliquot and the pH adjusted to 2.5 by addition of 1 M HCl. Digestions were then performed essentially as for the gliadin preparation; volumes of all additions were noted to account for dilution effects in subsequent analyses. Samples were placed on ice and then stored frozen at –20°C until required for further analysis.

Separate in vitro gastric digests were also performed in duplicate in the presence of phospholipid vesicles (6.3 mM) in highP SGF. For bread the oral‐gastric digestion was repeated using the “low” pepsin in SGF for 11 mins (G11) and the digest divided into 4 g aliquots and placed on ice prior to duodenal digestion.

*Duodenal digestion*: The pH of oral‐gastric digests was reduced to 2.5 by addition of 1 M HCl followed by addition of 0.68 mL/digestion aliquot of prewarmed (37°C) hepatic mix solution (HMS, 12.5 mM sodium taurocholate, 12.5 mM sodium glycodeoxycholate, 146 mM NaCl, 2.6 mM CaCl_2_, 4.8 mM KCl, 4 mM cholesterol) and 2 mL/aliquot of prewarmed (37°C) pancreatic mix solution (PMS, 0.6 mM CaCl_2_, 4.1 μM ZnSO_4_, 125 mM NaCl, 0.3 mM MgCl_2_) either alone (DU, duodenal undigested control) or containing trypsin (34.5 Nα‐benzoyl‐L‐arginine ethyl ester U/mg gliadin/flour/bread protein) chymotrypsin (11.8 *N*‐acetyl‐l‐tyrosine ethyl ester/mg gliadin/flour/bread protein) and pancreatic amylase (1.7 U/mg flour/bread carbohydrate). The pH was then raised to 6.5 with modified Krebs‐Ringer buffer (0.7 mM Na_2_HPO_4_, 0.49 mM MgCl_2_, 4.56 mM KCl, 1.5 mM NaH_2_PO_4_, 54.46 mM NaCl, 80.36 mM NaHCO_3_). Samples were incubated in a shaking incubator at 37°C, 170 rpm for either 0.3 min (DU only) or 0.3, 5, 15, 30, 60, and 120 min. Separate duodenal digests were performed omitting either the proteases or salivary and pancreatic amylase; using pancreatic amylase pretreated with 10 mM PMSF for 3 h at ambient temperature to inactivate residual proteases (duplicate only); or with the addition of lipase and colipase (8.4 U/mg four/bread fat, lipase: colipase added at 5:1 molar ratio). Proteolysis was stopped by addition of 1.2 mL of 0.1 M PMSF and samples then taken for starch and protein analysis, protein samples were placed on ice for 10 min prior to freezing at –20°C until required. The volumes of all additions were noted to account for dilution effects in subsequent analyses.

### 2.3 SDS‐PAGE and immunoblotting

Samples were thawed and centrifuged at 15 000 × *g* for 5 min at ambient temperature and the supernatant removed. To 50 μL of supernatant, 25 μL 200 mM DTT and 25 μL NuPAGE LDS buffer were added and samples were heated to 100°C for 15 min in a heat block. Pellets from total gliadin fraction (TGF) digests, or 10 mg undigested flour or bread digest were extracted in 7 M urea, 2 M thiourea, 2% w/v CHAPS, 50 mM DTT, pH 8.8 at 60°C in a sonicating water bath (3 × 5 min). After centrifugation at 10 000 × *g* for 5 min at ambient temperature the supernatants were removed and 25 μL NuPAGE LDS was added to 75 μL of supernatant prior to heating to 100°C for 15 min in a heat block. Samples were loaded onto 12% NuPAGE Bis‐tris gels together with either Mark 12^TM^ markers, or, for immunoblotting, SeeBlue^TM^ prestained markers. Electrophoresis was performed for 40 min at 200 V using NuPAGE 2‐(*N*‐modpholino)ethanesulfonic acid buffer (Invitrogen, UK). Gels were fixed for 2 h in 50% v/v methanol (MeOH), 10% v/v TCA and stained overnight with SimplyBlue^TM^ safestain prior to destaining with distilled water and imaging using a Typhoon Trio scanner (GE Healthcare, Buckinghamshire, UK). Protein analysis was performed for each oral‐gastric digest and for two out of the three oral‐gastro‐duodenal digests, giving two sets of SDS‐PAGE and immunoblot data for each in vitro digestion condition.

For immunoblotting, SDS‐PAGE gels were soaked in 25 mM Tris‐HCl pH 8.3 containing 125 mM glycine, 20% v/v MeOH for 20 mins. Electro‐blotting was performed with nitrocellulose membrane (Biorad, Hertfordshire, UK) using a semidry blotting system (Biorad) at 20 V for 25 min. Membranes were washed for 10 min in PBS (2 mM NaH_2_PO_4_, 8 mM Na_2_HPO_4_, 0.58 M NaCl, pH 7.4), 0.05% v/v Tween 20 (wash buffer) then blocked overnight at 4°C in wash buffer containing 5% w/v skimmed milk powder (blocking buffer). Membranes were then washed for 4 × 5 min with wash buffer and incubated for 1 h at ambient temperature with mAb IFRN 0610 or IFRN 0065 culture supernatant diluted 1:100 v/v in blocking buffer 30. The 4 × 5 min washing step was repeated for membranes before incubating with secondary anti‐mouse IgG labeled with alkaline phosphatase for 1 h, diluted 1:5000 v/v in blocking buffer. Membranes were washed for 4 × 5 min with wash buffer then developed for 15 min using NBT/BCIP substrate solution diluted 1:2 v/v in water. Finally, blots were rinsed with water and dried prior to imaging using a charged couple device camera with four orders of magnitude linearity (Fujifilm LAS‐1000, Fuji, Japan).

### 2.4 Densitometric analysis

Selected polypeptides that were reproducibly resolved as discrete bands on duplicate immunoblots were subjected to densitometric analysis using ImageQuant software (GE Healthcare, Buckinghamshire, UK). Densitometry was performed using the rolling ball method for background subtraction and linlog standard curve with molecular weight marker proteins as standards for semiquantification. The averaged intensity of each band from duplicate blots was normalized to “GU” or 120 min for substrate or product polypeptides, respectively. Normalized intensities were plotted as a function of digestion time using an exponential curve fit corresponding to a first‐order reaction 31 with models built in R software (http://www.r‐project.org/) using Nelder–Mead for nonlinear optimization, producing rate constants (k, min^−1^) for each protein. “GU” was used as a hypothetical “0 min” time point.

### 2.5 Starch analysis

Bread digests were sampled at each time point for starch analysis by adding 5 mL ice‐cold 80% v/v ethanol to aliquots of the chew, gastric (∼100 mg wet weight) or duodenal (∼300 mg wet weight) digests. Subsequently, samples were heated at 84°C for 6 min before analysis using the total starch assay kit on insoluble starch (Megazyme, UK). A two‐way ANOVA followed by bonferroni post‐hoc tests were performed on starch data using GraphPad Prism version5.04 for Windows (GraphPad Software, California, USA, www.graphpad.comPRISM).

## 3 Results

The effect of baking on the digestibility of wheat flour proteins was investigated using a batch model of digestion that simulated conditions in the oral cavity, stomach, and duodenum. Gastric digestion was undertaken using two different concentrations of pepsin, the higher of which had previously been used in digestion studies of purified proteins 12, 25.

**Figure 1:**
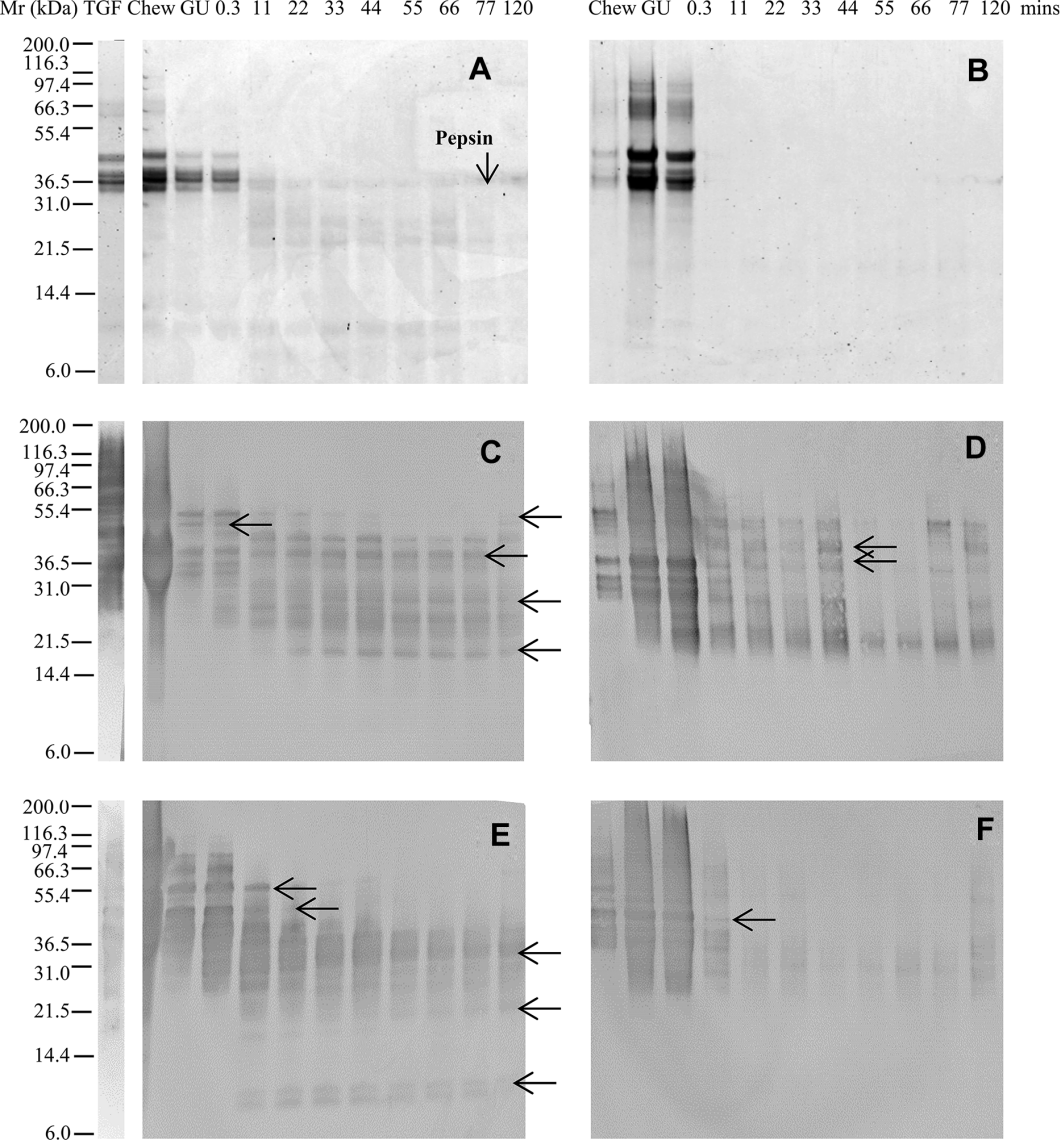
Simulated gastric digestion of a total gliadin fraction from wheat undertaken using a low pepsin protocol. Soluble (A, C, E) and insoluble (B, D, F) fractions of digests analysed by SDS‐PAGE (A, B), immunoblotting (C–F) using mAbs IFRN 0610 (C, D; brightened by + 40%) and 0065 (E, F). Selected bands (arrowed) were subjected to densitometric analysis (Supporting Information Table 3) and used for kinetic analysis. Soluble protein volumes loaded accounted for dilution factor between digestive phases whereas extracted insoluble protein samples were loaded in equivalent volumes. Pepsin was identified on the basis of its Mr defined by SDS PAGE analysis of the digestion enzymes (data not shown).

To aid the interpretation of proteolysis in such complex mixtures a purified total gliadin preparation (TGF, cv. Hereward) was initially subjected to digestion using only the oral‐gastric stage of the models. The TGF comprised a mixture of proteins of M_r_ 35 000–45 000 Da (Fig. 1A), much of which was soluble in the model salivary fluid used in the model chew. However, on addition of SGF the TGF largely precipitated (Chew, GU, Fig. 1A, B), although both the soluble and insoluble gliadin polypeptides were rapidly digested, with just a trace of poorly staining material remaining after 11 min digestion (Fig. 1B; Supporting Information Fig. 1B). Immunoreactive polypeptides were identified by immunoblotting much longer into the digestion time course (Fig. 1C–F and Supporting Information Fig. 1C–F). This is partly because Coomassie brilliant blue G‐250 binds less well to gluten because it is deficient in basic amino acids to which the dye binds 31, 32, 33 together with the fact the mAbs bind to gluten proteins with high affinity. The mAb IFRN 0610, which is specific for gliadins and LMW glutenins 30, only recognized digestion products down to M_r_ ∼20 000 Da (Fig. 1 and Supporting Information Fig. 1C, D) while a second mAb of similar specificity (IFRN 0065) recognized smaller fragments of down to M_r_ ∼6000 Da (Fig. 1 and Supporting Information Fig. 1E, F). In general, both digestion models gave qualitatively similar patterns of immunoreactive polypeptides for each mAb, with the disappearance of original “parent” proteins associated with the generation of “daughter” peptides resulting from the action of pepsin, some of which were transient in nature.

**Figure 2:**
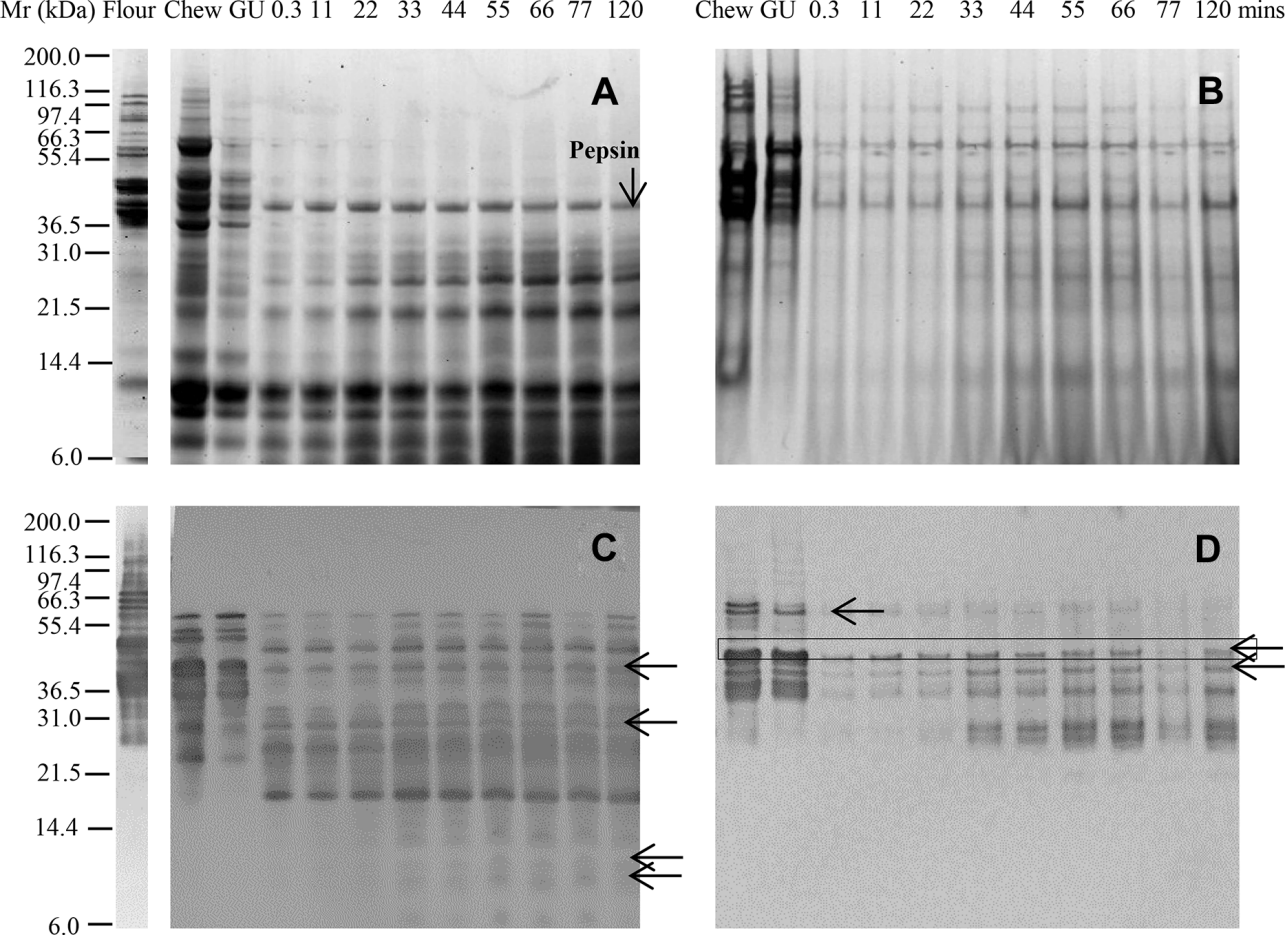
Simulated oral‐gastric digestion of wheat flour protein undertaken using a low pepsin protocol. Soluble (A, C) and insoluble (B, D) fractions of digests were analyzed by SDS‐PAGE (A, B) and immunoblotting (C, D) using mAb IFRN 0610 (C, D; brightened by + 40%). Selected bands (arrowed) were subjected to densitometric analysis (Supporting Information Table 3) and used for kinetic analysis. Soluble protein volumes loaded accounted for dilution factor between digestive phases whereas extracted insoluble protein samples were loaded in equivalent volumes. Pepsin was identified on the basis of its M_r_ defined by SDS PAGE analysis of the digestion enzymes (data not shown).

This approach was then applied to studying the digestibility of gluten proteins in flour using the “low” pepsin model only (Fig. 2). A complex mixture of proteins was solubilized in the SSF, including prominent lower M_r_ polypeptides likely to be α‐amylase inhibitors (Fig. 2A), identified based on M_r_ and immunoblotting with antibody specific to α‐amylase inhibitor CM3, donated by J Marsh (data not shown) 34, 35. Some prolamins were also solubilized, as indicated by the immunoreactivity with IFRN 0610 and 0065 (Fig. 2C, Supporting Information Fig. 2A). However, a substantial proportion of the protein remained in the pellet (Fig. 2B), addition of SGF resulting in precipitation of one prominent M_r_ 55 000 Da protein solubilized in the SSF. As gastric digestion proceeded, some of the SSF soluble polypeptides were rapidly digested including M_r_ 42 000 and 35 000 Da components, while the M_r_ 8000–12 000 Da polypeptides remained undigested for the entire 120 min gastric digestion time course. A broad range of M_r_ 9000–65 000 Da immunoreactive gluten proteins and their digestion products were observed on the immunoblots (Fig. 2C, D, Supporting Information Fig. 2A, B) that formed a number of “daughter” polypeptides of M_r_ 20 000–32 000 Da, which reached a steady‐state end‐point within the 120 min of the digestion time course. Polypeptides were rapidly lost from the insoluble fraction (Fig. 2B, 0.3–11 min) although a residual, faintly staining pattern of polypeptides remained undigested including two clearly resolved polypeptides of M_r_ 40 000 and 60 000 Da.

**Figure 3:**
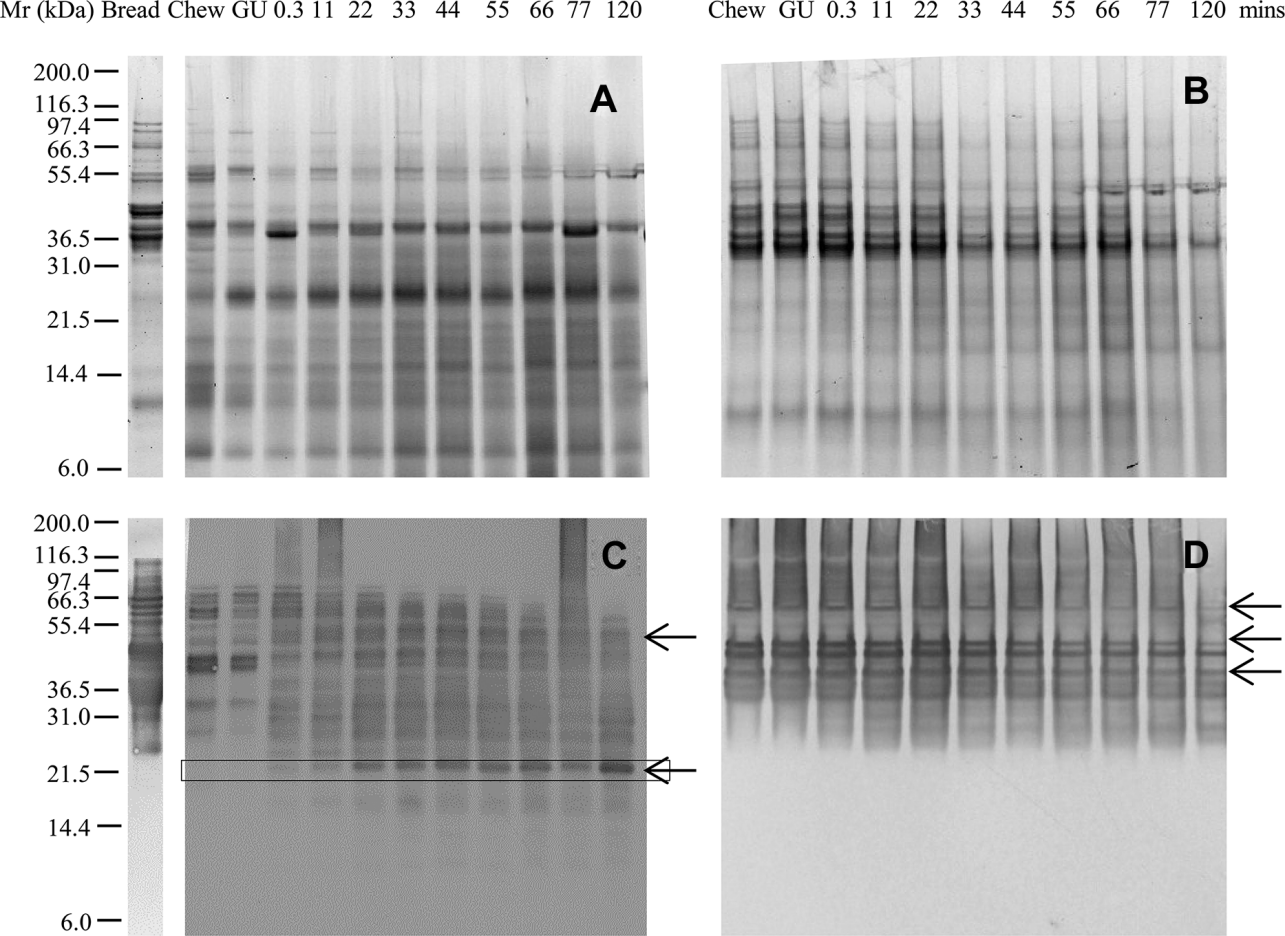
Simulated oral‐gastric digestion of bread protein undertaken using a low pepsin protocol. Soluble (A, C) and insoluble (B, D) fractions of digests were analyzed by SDS‐PAGE (A, B) and immunoblotting (C, D) using mAb IFRN 0610 (C, D brightened by + 40%). Selected bands (arrowed) were subjected to densitometric analysis (Supporting Information Table 3) and used for kinetic analysis. Soluble protein volumes loaded accounted for dilution factor between digestive phases whereas extracted insoluble protein samples were loaded in equivalent volumes.

Baking reduced the solubility of the gluten proteins, which were generally less well resolved and were more resistant to proteolysis in bread than in flour (Fig. 3A, B and Supporting Information Fig. 3A, B). This was confirmed by immunoblotting with IFRN 0610 and 0065 (Fig. 3C, D, Supporting Information Fig. 2C, D) which revealed a complex pattern of immunoreactive polypeptides, a number of which, such as a M_r_ 20 000 Da polypeptide, remained even after 120 min gastric digestion (Fig. 3C). Inclusion of phospholipid vesicles in the gastric digestion model did not affect either the pattern or kinetics of digestion (Supporting Information Fig. 4).

**Figure 4:**
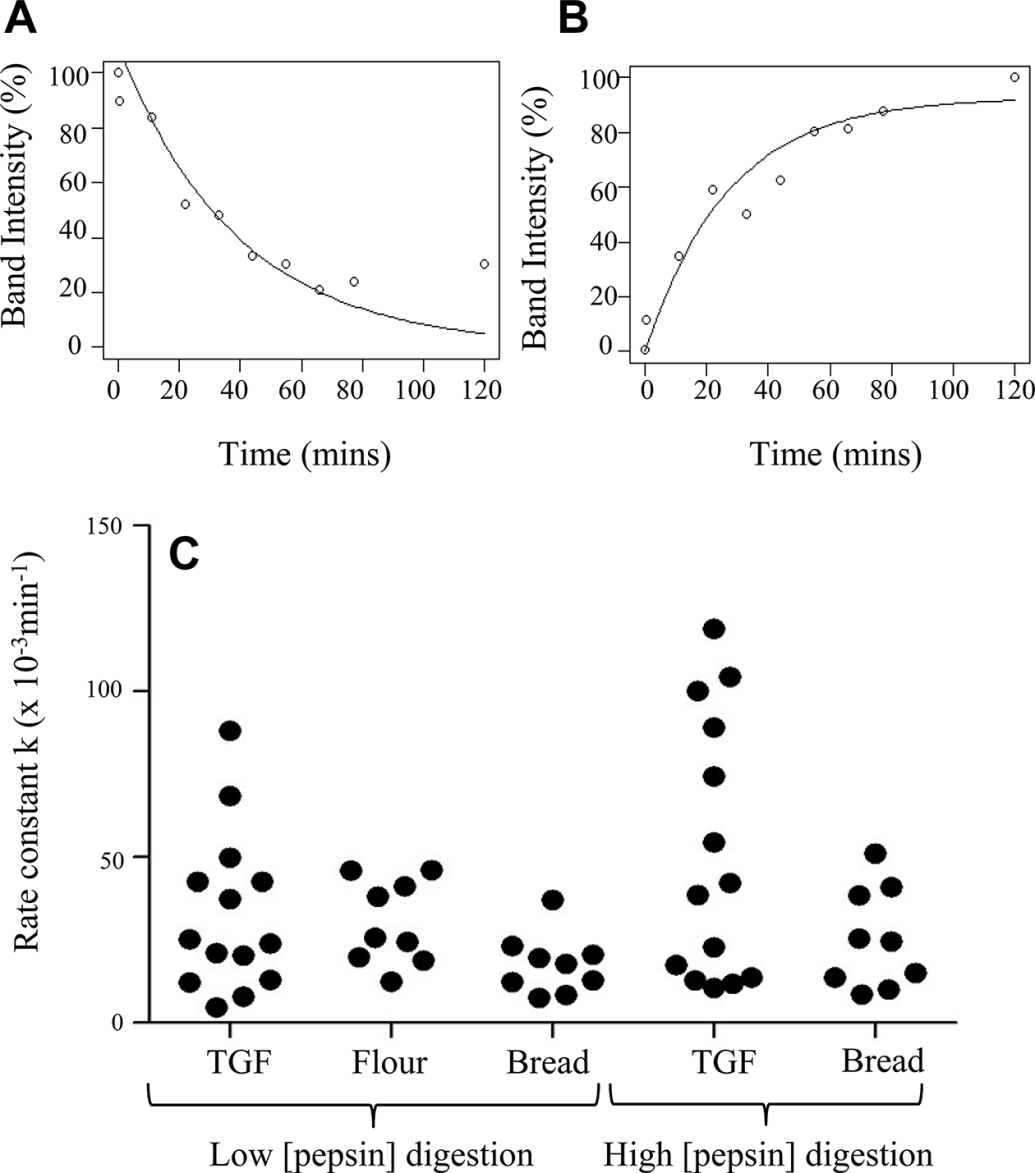
Kinetics of selected prolamins under different digestion conditions. Kinetics (rate constant *k* (min^−1^) estimated from the fitting of an exponential curve) of the disappearance of substrate protein or appearance of product at “high” or “low” pepsin: protein ratios. Prolamins were reactive with mAb 0610 or 0065, present in digest immunoblots of matrices total gliadin fraction (TGF), flour or bread. (A) Kinetic analysis example from flour digestion, M_r_ 44 kDa insoluble substrate, *k* = 25.66 × 10^−3^min^−1^. (B) Kinetic analysis example from bread digestion (low pepsin), M_r_ 20 kDa soluble product, *k* = 37.09 × 10^−3^min^−1^. (C) Scatter plot of combined kinetic analysis for digests.

Densitometric analysis of immunoblots was used to semiquantify the digestion patterns of between 9 and 14 polypeptides recognized in each sample by either mAb 0610 or 0065. Data were of sufficient quality to allow calculation of first‐order rate constants (Fig. 4 and Supporting Information Table 3). Different types of digestion behavior were observed. Some polypeptides were rapidly digested, such as a M_r_ 44 000 Da band in the insoluble fraction of flour (Fig. 2D, kinetics shown in Fig. 4A). In other cases, stable fragments were released that accumulated over time, such as a M_r_ 20 000 Da polypeptide in bread that gave a rate constant of 37.09 × 10^−3^ min^−1^ (Fig. 3C, kinetics shown in Fig. 4B). Using the low pepsin concentration, rate constants for polypeptides from the TGF rates spanned a wider range (4.74–104.35 × 10^−3^min^−1^) and were generally higher than for polypeptides present in the flour and bread samples, both of which had k values ranging within 7.59–51.05 × 10^−3^min^−1^ (Fig. 4, Supporting Information Table 3). A similar pattern was observed when a high pepsin concentration was used for in vitro digestion (Fig. 4, Supporting Information Table 3). Rate constants for polypeptides in the bread digests were largely the same irrespective of the level of inclusion of pepsin, suggesting that even for the “low” pepsin conditions, the enzyme was not rate limiting.

### 3.1 Simulated gastroduodenal digestion

**Figure 5:**
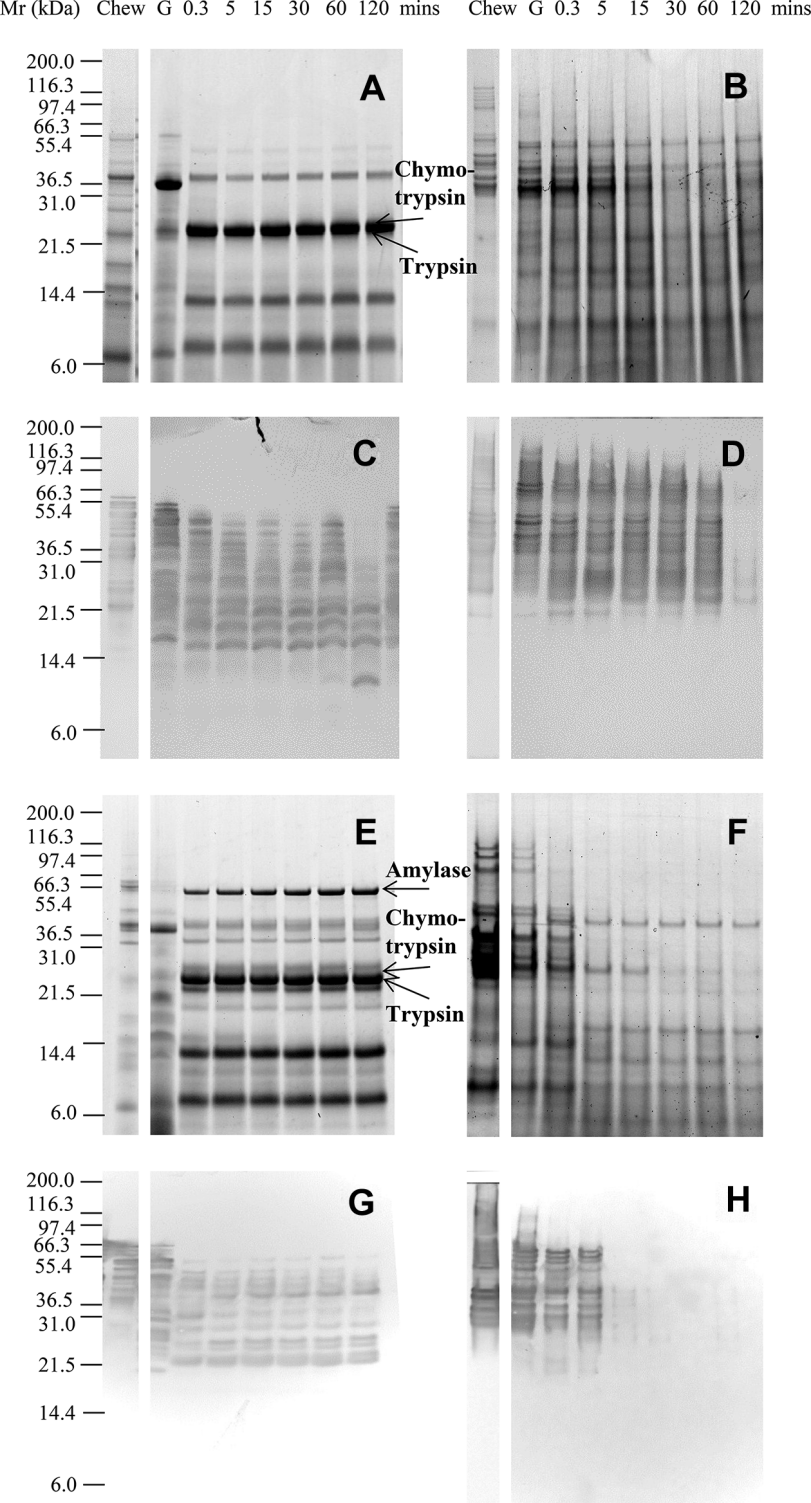
Simulated gastro‐duodenal digestion of protein from bread. SDS‐PAGE (A–D) and immunoblots (E–H) from oral, gastric, and duodenal/intestinal digestion of bread after 11 min low‐pepsin gastric digestion. Performed without (A, B, E, F) or with (C, D, G, H) HSA and PMSF‐inhibited pancreatic amylase present. Soluble (A, C, E, G) and insoluble (B, D, F, H) fractions were recovered by centrifugation. Immunoblots developed using mAb 0610 and brightened up to + 40%. Soluble protein volumes loaded accounted for dilution factor between digestive phases whereas extracted insoluble protein samples were loaded in equivalent volumes. Pancreatic α‐amylase, chymotrypsin, and trypsin were identified on the basis of its M_r_ defined by SDS PAGE analysis of the digestion enzymes (data not shown).

**Figure 6:**
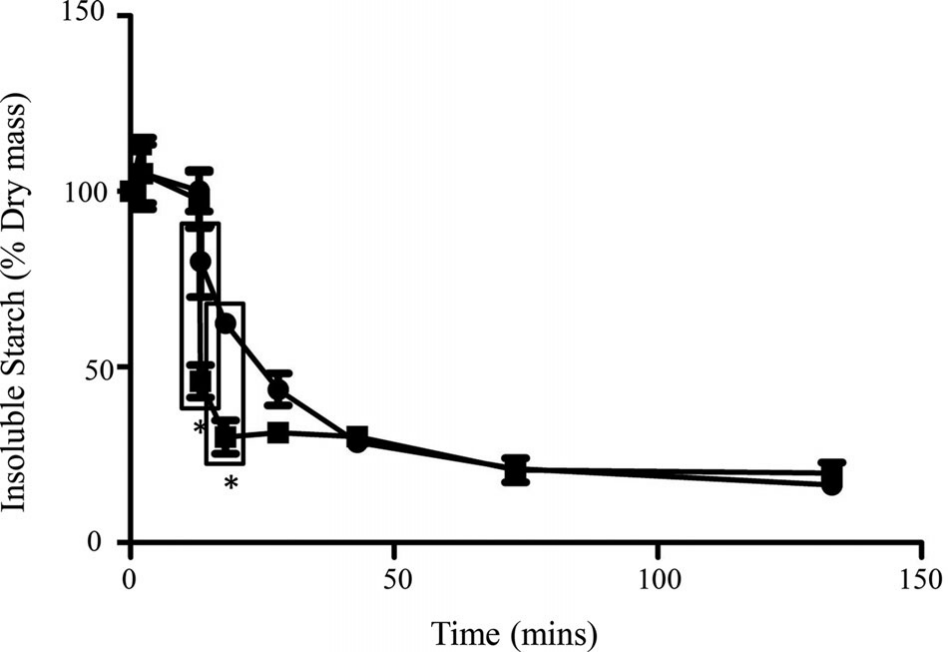
Breakdown of starch in bread samples during simulated gastro‐duodenal digestion. Insoluble starch as % dry matter at different time points of digestion. “0” refers to “chew” sample, with time points onwards as 0.3, 11 min gastric digestion and duodenal time points. Two digestion conditions plotted: 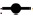 with amylases only 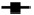 with amylases and proteases present. *Statistically significant data points (G11D0.3 *p* < 0.001, G11D5 *p* < 0.01). Bars show standard error of the mean.

Gastric digests of bread from the 11 min time point were then subjected to simulated duodenal digestion with or without amylases included in the duodenal digestion medium (Fig. 5, Supporting Information Fig. 5, 6); this was not undertaken for the TGF or flour as insufficient protein remained after gastric digestion. In the absence of amylases, gluten proteins were slowly digested in the soluble fraction, with highly immunoreactive insoluble proteins persisting throughout the time course of duodenal digestion (Fig. 5A–D). Inclusion of amylases in the digestion protocol resulted in gluten proteins being more rapidly solubilized and digested by pancreatic endoproteases, with polypeptides of M_r_ 40–55 000 Da rapidly decreasing in intensity after 5 min duodenal digestion (Fig. 5F, H; Supporting Information Fig. 5D). Immunoblots using IFRN 0610 showed that immunoreactive gluten protein was solubilized after 5–15 min digestion and remained detectable in the soluble fraction even after 120 min duodenal digestion (Fig. 5G). Proteolysis also affected the rate of starch digestion with the loss of insoluble starch being slower in the digest carried out without proteases especially early during the duodenal digestion time course (Fig. 6). Thus, statistically significant differences in starch digestion were observed between digests undertaken with and without proteases after 0.3 (*p* < 0.001) and 5 min (*p* < 0.01) of duodenal digestion.

## 4 Discussion and conclusion

The form of the gluten proteins had a marked effect on their digestibility, with the purified protein fraction (TGF) being rapidly digested, the flour proteins being digested more slowly, and the bread proteins being resistant to digestion. The current study shows that while more precise information on the mechanisms of digestion can be gained from studying purified proteins, such data are not predictive of digestion in complex food matrices and may be misleading. The greater digestibility of flour compared to bread is likely due to the particulate nature of flour having a greater surface area and hence providing digestive enzymes with greater accessibility to protein or starch. Others have previously observed that bread and pasta are digested only slowly 24, 36.

Many of the gluten proteins in bread were highly resistant to digestion, remaining even after 120 min of simulated gastric digestion even when a high pepsin concentration was employed. This included proteins that contained QQSF and QPFP epitopes recognized by the mAbs IFRN 0610 and 0065, which are present in the highly celiac‐toxic γ36 6 and γ5 gliadins 37, respectively. This indicates that the pepsin concentration is not rate limiting for bread digestion in this system, and it is probable that the protein is digested slowly because it is poorly accessible to pepsin. After baking, starch granules and protein are likely to be less accessible to digestive enzymes than in flour, as a result of a network of aggregated proteins in which starch granules and lipid inclusions are embedded. Furthermore, chemical modification of proteins as a result of baking, notably resulting from Maillard‐type reactions, may also modulate digestibility, although only to a limited extent due to the lack of lysine residues in many gluten polypeptides. Such processing‐induced changes will be compounded by the fact the bread matrix forms a bolus during chewing with a much smaller surface area to volume ratio than the flour. This is likely to slow the ingress of digestive fluids into the bolus, and further reduce digestive enzyme accessibility to protein or starch.

The observation that the rate of protein digestion in bread increased in the presence of amylases probably results from the accessibility to gluten proteins being increased as a consequence of breakdown of the starch granules and any amylose that may have leached out from them during the baking process. Proteolysis of the gluten network also enhanced starch digestion, presumably because degradation of the gluten network in which the starch granules are embedded improves accessibility of amylases to the granule surfaces. These observations highlight how digestion is a synergistic process, with different digestive enzymes working in concert to disrupt a complex matrix. Thus, the protein structure will contribute to determining the glycaemic index of foods, while interactions between the protein and starch factions will modify protein digestibility. The interdependence of starch and protein digestibility in complex food matrices such as bread could have important implications for patients who are deficient in pancreatic amylase 38 and may offer insights into how foods might be formulated that would allow more effective protein digestion by such individuals. Low levels of pancreatic amylase secretion are common in infancy and early childhood 39 and could impact on protein digestion of starch‐rich foods in such individuals. The maturity of the gastrointestinal tract may play an important role in determining susceptibility to developing immune‐mediated adverse reactions to foods, firstly by affecting how food proteins are presented to the body as a consequence of the digestive process, and secondly how the gut mucosal immune system responds to them. Future work will build on the results presented here, obtained using a batch model of digestion, and extend them to assess how the baking process may affect digestibility using a dynamic model of digestion that takes account of both the gastric and duodenal compartments 27, 28. Such dynamic models provide a more realistic simulation of the physical aspects of the digestive process, which are important when taking into account the effects of the food matrix on digestion 40.

*The authors have declared no conflict of interest*.

## Supporting information

As a service to our authors and readers, this journal provides supporting information supplied by the authors. Such materials are peer reviewed and may be re‐organized for online delivery, but are not copy‐edited or typeset. Technical support issues arising from supporting information (other than missing files) should be addressed to the authors.

Supporting Figure
Supporting Table
