# Supplementary material for: Digestibility of gluten proteins is reduced by baking and enhanced by starch digestion
Source: Mol Nutr Food Res. 2015 Aug 21;59(10):2034–43. doi: 10.1002/mnfr.201500262 (PMC4949995; doi:10.1002/mnfr.201500262)
Supplement: Supplementary file 1 — Supporting Figure Supporting Table [file MNFR-59-2034-s001.zip › mnfr2453-sup-0002-FigureS2.docx]

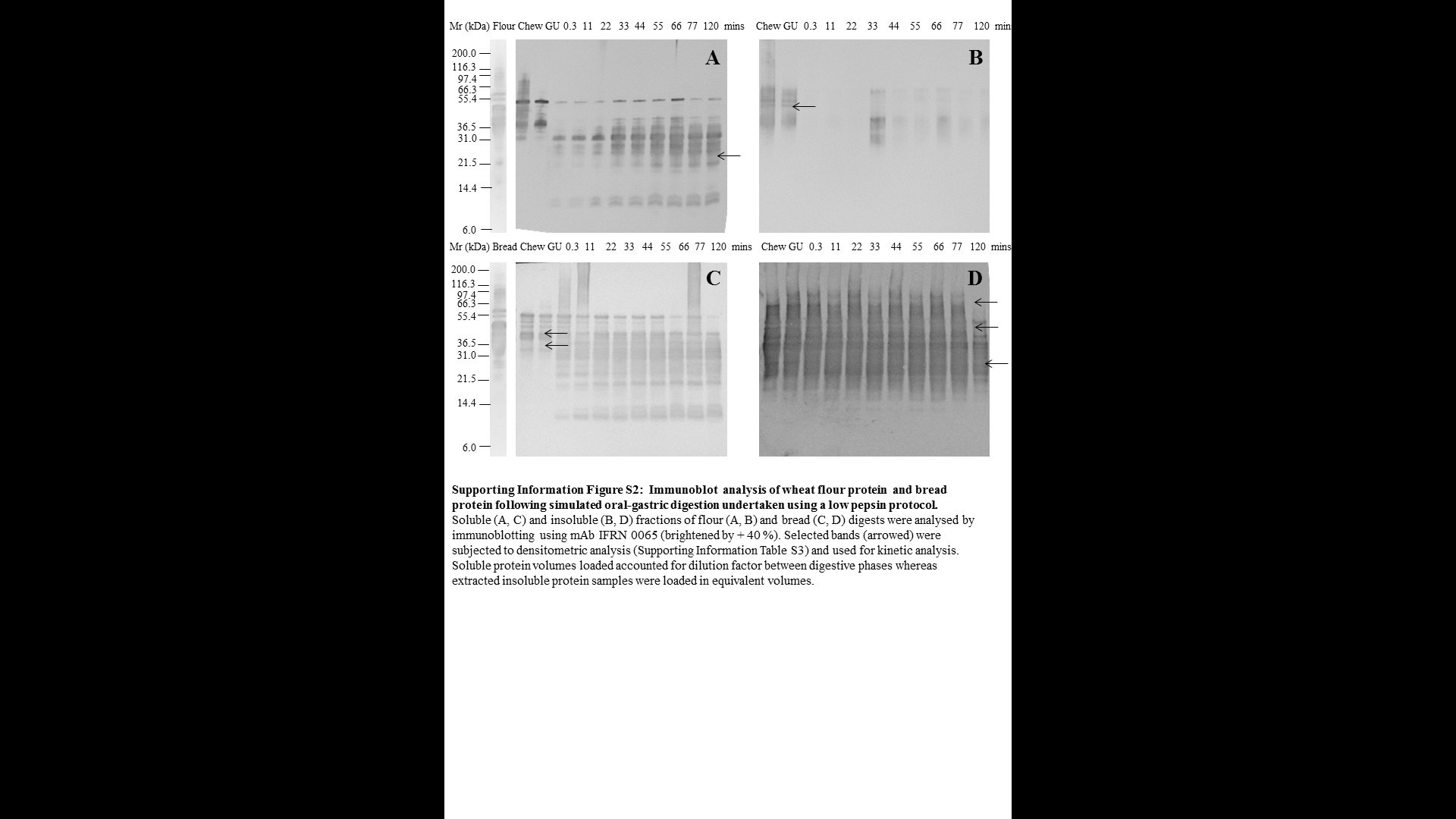


**Supporting Information Figure S2: Immunoblot analysis of wheat flour protein and bread protein following simulated oral-gastric digestion undertaken using a low pepsin protocol.**

Soluble (A, C) and insoluble (B, D) fractions of flour (A, B) and bread (C, D) digests were analysed by immunoblotting using mAb IFRN 0065 (brightened by + 40 %). Selected bands (arrowed) were subjected to densitometric analysis (Supporting Information Table S3) and used for kinetic analysis. Soluble protein volumes loaded accounted for dilution factor between digestive phases whereas extracted insoluble protein samples were loaded in equivalent volumes.
