# Supplementary material for: Digestibility of gluten proteins is reduced by baking and enhanced by starch digestion
Source: Mol Nutr Food Res. 2015 Aug 21;59(10):2034–43. doi: 10.1002/mnfr.201500262 (PMC4949995; doi:10.1002/mnfr.201500262)
Supplement: Supplementary file 1 — Supporting Figure Supporting Table [file MNFR-59-2034-s001.zip › mnfr2453-sup-0004-FigureS4.docx]

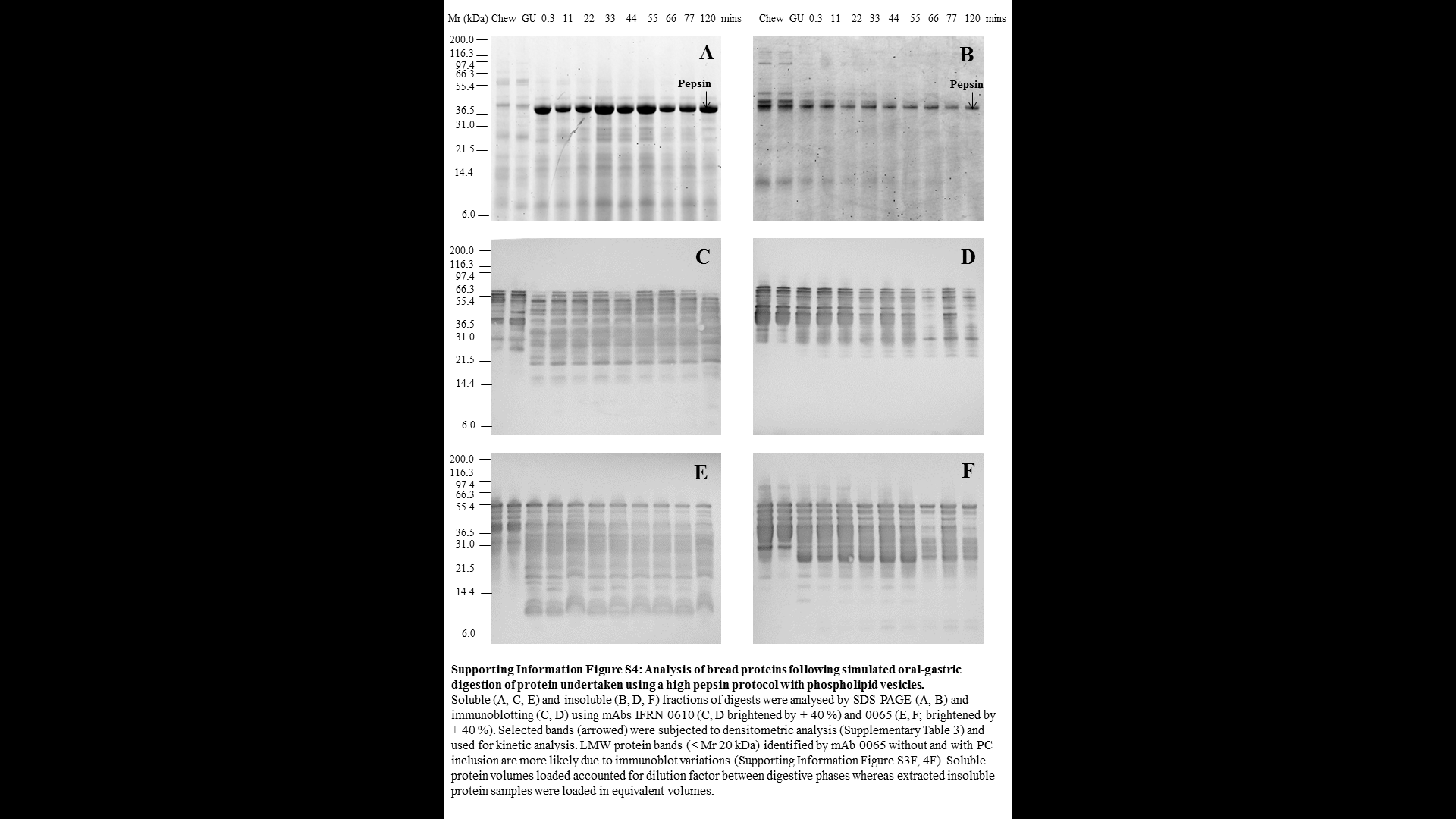


**Supporting Information Figure S4: Analysis of bread proteins following simulated oral-gastric digestion of protein undertaken using a high pepsin protocol with phospholipid vesicles.**

Soluble (A, C, E) and insoluble (B, D, F) fractions of digests were analysed by SDS-PAGE (A, B) and immunoblotting (C, D) using mAbs IFRN 0610 (C, D brightened by + 40 %) and 0065 (E, F; brightened by + 40 %). Selected bands (arrowed) were subjected to densitometric analysis (Supplementary Table 3) and used for kinetic analysis. LMW protein bands (< Mr 20 kDa) identified by mAb 0065 without and with PC inclusion are more likely due to immunoblot variations (Supporting Information Figure S3F, 4F). Soluble protein volumes loaded accounted for dilution factor between digestive phases whereas extracted insoluble protein samples were loaded in equivalent volumes.
