# Supplementary material for: Digestibility of gluten proteins is reduced by baking and enhanced by starch digestion
Source: Mol Nutr Food Res. 2015 Aug 21;59(10):2034–43. doi: 10.1002/mnfr.201500262 (PMC4949995; doi:10.1002/mnfr.201500262)
Supplement: Supplementary file 1 — Supporting Figure Supporting Table [file MNFR-59-2034-s001.zip › mnfr2453-sup-0005-FigureS5.docx]

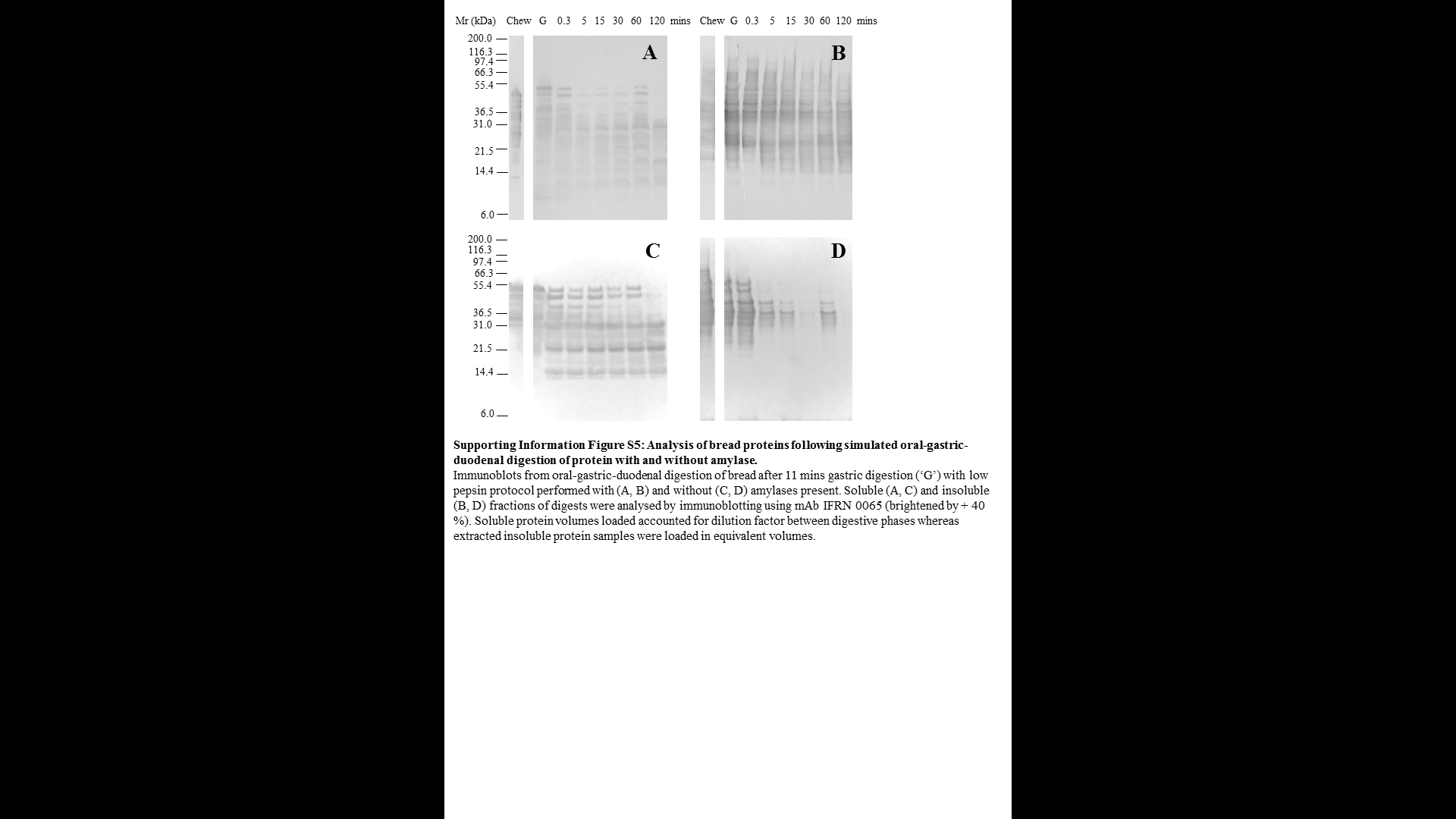


**Supporting Information Figure S5: Analysis of bread proteins following simulated oral-gastric-duodenal digestion of protein with and without amylase.**

Immunoblots from oral-gastric-duodenal digestion of bread after 11 mins gastric digestion (‘G’) with low pepsin protocol performed with (A, B) and without (C, D) amylases present. Soluble (A, C) and insoluble (B, D) fractions of digests were analysed by immunoblotting using mAb IFRN 0065 (brightened by + 40 %). Soluble protein volumes loaded accounted for dilution factor between digestive phases whereas extracted insoluble protein samples were loaded in equivalent volumes.
