# Supplementary material for: Digestibility of gluten proteins is reduced by baking and enhanced by starch digestion
Source: Mol Nutr Food Res. 2015 Aug 21;59(10):2034–43. doi: 10.1002/mnfr.201500262 (PMC4949995; doi:10.1002/mnfr.201500262)
Supplement: Supplementary file 1 — Supporting Figure Supporting Table [file MNFR-59-2034-s001.zip › mnfr2453-sup-0006-FigureS6.docx]

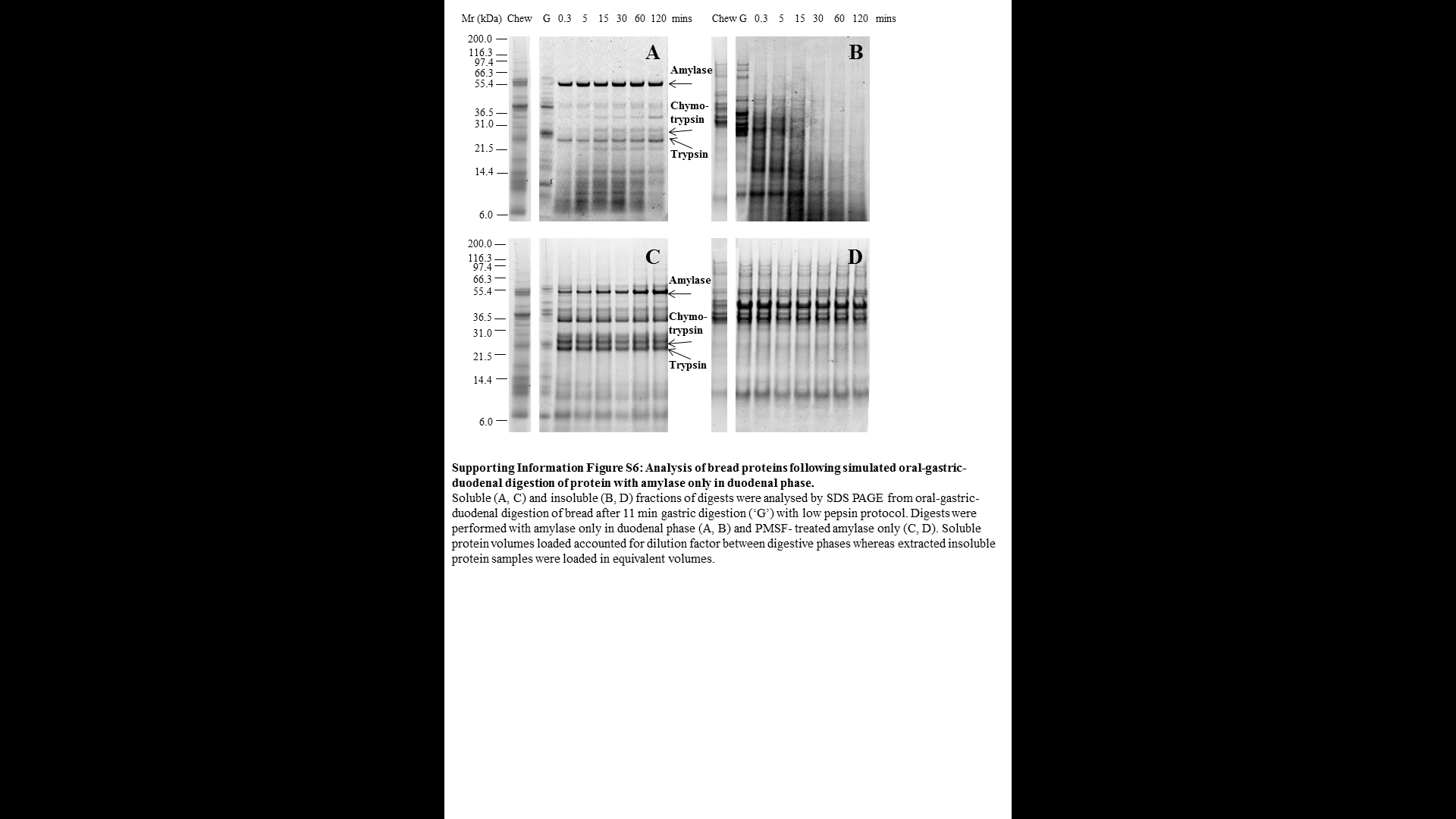


**Supporting Information Figure S6: Analysis of bread proteins following simulated oral-gastric-duodenal digestion of protein with amylase only in duodenal phase.**

Soluble (A, C) and insoluble (B, D) fractions of digests were analysed by SDS PAGE from oral-gastric-duodenal digestion of bread after 11 min gastric digestion (‘G’) with low pepsin protocol. Digests were performed with amylase only in duodenal phase (A, B) and PMSF- treated amylase only (C, D). Soluble protein volumes loaded accounted for dilution factor between digestive phases whereas extracted insoluble protein samples were loaded in equivalent volumes.
