# Supplementary material for: Digestibility of gluten proteins is reduced by baking and enhanced by starch digestion
Source: Mol Nutr Food Res. 2015 Aug 21;59(10):2034–43. doi: 10.1002/mnfr.201500262 (PMC4949995; doi:10.1002/mnfr.201500262)
Supplement: Supplementary file 1 — Supporting Figure Supporting Table [file MNFR-59-2034-s001.zip › mnfr2453-sup-0007-TableS1.docx]

**Supporting Information Table S1: Enzymes used in simulated *in vitro* digestion studies and their activities**

| **Enzyme** | **Source** | **Supplier** | **Activity (U/mg)** | **Unit definition** |
| --- | --- | --- | --- | --- |
| Salivary amylase (HSA) | Human | Applichem GmbH (Kongens Lyngby, Denmark) | 126 | One unit will result in the formation of 5µM glucose 6-phosphate/min through coupled reactions with nicotinamide adenine dinucleotide at 37^o^C, monitored at 340 nm, using maltopentaose as substrate |
| Pepsin | Porcine | Sigma-Aldrich (Dorset, UK) | 4293 | One unit will produce a change in absorbance at 280 nm of 0.001/min at pH 2.0 and 37^o^C, measured as trichloroacetic acid (TCA) soluble products using haemoglobin as substrate |
| α-amylase | Porcine | Sigma-Aldrich (Dorset, UK) | 50 | One unit will liberate 1 µmol maltose/min at pH 6.9 and 25^o^C, with potato starch as substrate |
| Trypsin | Porcine | Applichem GmbH (Kongens Lyngby, Denmark) | 780 | One unit will produce a change in absorbance at 253 nm of 0.001/min at pH 7.6 and 25^o^C using Nα-Benzoyl-L-Arginine Ethyl Ester (BAEE) as substrate |
| α-chymotrypsin | Bovine | Merck (Nottinghamshire, UK) | 350 | One unit will produce a change in absorbance at 237 nm of 0.0075/min at pH 7.0 and 25^o^C using N-acetyl-L-tyrosine ethyl ester (ATEE) as substrate |
| Lipase and colipase | Porcine | MP Biomedicals (Illkirch Cedex, France), Sigma-Aldrich (Dorset, UK) | 25200  (5:1 molar ratio lipase:colipase) | One unit will liberate 100 µM fatty acid/h at pH 7.8 and 37^o^C using olive oil emulsion as substrate |
