# Supplementary material for: Digestibility of gluten proteins is reduced by baking and enhanced by starch digestion
Source: Mol Nutr Food Res. 2015 Aug 21;59(10):2034–43. doi: 10.1002/mnfr.201500262 (PMC4949995; doi:10.1002/mnfr.201500262)
Supplement: Supplementary file 1 — Supporting Figure Supporting Table [file MNFR-59-2034-s001.zip › mnfr2453-sup-0008-TableS2.docx]

**Supporting Information Table S2:** **Details of components in molecular markers used for SDS-PAGE.**

| **Marker Type** | **Protein** | **Mr (kDa)** |
| --- | --- | --- |
| Mark 12^TM^ marker (Invitrogen) | Myosin | 200.0 |
|  | β-galactosidase | 116.3 |
|  | Phosphorylase b | 97.4 |
|  | Bovine serum albumin (BSA) | 66.3 |
|  | Glutamic dehydrogenase | 55.4 |
|  | Lactate dehydrogenase | 36.5 |
|  | Carbonic anhydrase | 31 |
|  | Trypsin inhibitor | 21.5 |
|  | Lysozyme | 14.4 |
|  | Apoprotinin | 6 |
|  | Insulin B chain | 3.5 |
|  | Insulin A chain | 2.5 |
| SeeBlue^TM^ prestained marker  (Invitrogen) | Myosin | 188 |
|  | Bovine serum albumin (BSA) | 62 |
|  | Glutamic dehydrogenase | 49 |
|  | Alcohol dehydrogenase | 38 |
|  | Carbonic anhydrase | 28 |
|  | Myoglobin | 18 |
|  | Lysozyme | 14 |
|  | Apoprotinin | 6 |
|  | Insulin B chain | 3 |
