# Supplementary material for: Digestibility of gluten proteins is reduced by baking and enhanced by starch digestion
Source: Mol Nutr Food Res. 2015 Aug 21;59(10):2034–43. doi: 10.1002/mnfr.201500262 (PMC4949995; doi:10.1002/mnfr.201500262)
Supplement: Supplementary file 1 — Supporting Figure Supporting Table [file MNFR-59-2034-s001.zip › mnfr2453-sup-0009-TableS3.docx]

**Supporting Information Table S3: Kinetics of prolamins under different gastric digestion conditions.**

Kinetics (rate constant k (min^-1^) estimated from the fitting of an exponential curve) of the disappearance or appearance of substrate or product protein/polypeptide at ‘high’ and ‘low’ pepsin: protein ratios. Prolamins were reactive with mAb IFRN 0610 or 065, present in digest immunoblots of matrices total gliadin fraction (TGF), flour or bread. Corresponding blot data is referenced.

| **Sample** | **Fraction** | **mAb** | **Type** | **Polypeptide Mr (kDa)** | **First order rate constant k (x 10^-3^min^-1^)** | | **Figure** |
| --- | --- | --- | --- | --- | --- | --- | --- |
|  |  |  |  |  | **“low” pepsin** | **“high” pepsin** |  |
| Total gliadin fraction | Soluble | 0610 | Substrate | 61 | 37.34 | 17.44 | 1C, Supporting Information Figure S1C |
|  |  |  |  | 50 | 4.74 | 118.81 |  |
|  |  |  |  | 46 | 7.95 | 74.33 |  |
|  |  |  | Product | 27 | 49.78 | 42.24 |  |
|  |  |  |  | 20 | 88.10 | 54.49 |  |
|  |  | 0065 | Substrate | 58 | 42.57 | 100.00 | 1E, Supporting Information Figure S1E |
|  |  |  |  | 48 | 25.18 | 22.85 |  |
|  |  |  | Product | 33 | 68.39 | 104.35 | 1E, Supporting Information Figure S1E |
|  |  |  |  | 21 | 23.98 | 38.60 |  |
|  |  |  |  | 11 | 42.51 | 89.12 |  |
|  | Insoluble | 0610 | Substrate | 46 | 12.22 | 10.56 | 1D, Supporting Information Figure S1D |
|  |  |  |  | 40 | 12.91 | 13.66 |  |
|  |  |  |  | 37 | 20.34 | 12.75 |  |
| Total Gliadin Fraction | Insoluble | 0065 | Substrate | 47 | 21.09 | 11.82 | 1F, Supporting Information Figure S1F |
| Flour | Soluble | 0610 | Substrate | 44 | 24.42 | - | 2C |
|  |  |  |  | 30 | 12.45 | - | 2C |
|  |  |  | Product | 11 | 38.11 | - |  |
|  |  |  |  | 9 | 46.06 | - |  |
|  |  | 0065 | Product | 22 | 18.75 | - | Supporting Information Figure S2A |
|  | Insoluble | 0610 | Substrate | 61 | 19.82 | - | 2D |
|  |  |  |  | 44 | 25.66 | - |  |
|  |  |  |  | 41 | 41.21 | - |  |
|  |  | 0065 |  | 51 | 45.88 | - | Supporting Information Figure S2B |
| Bread | Soluble | 0610 | Substrate | 45 | 12.37 | 38.45 | 3C, Supporting Information Figure S2C |
|  |  | 0065 |  | 40 | 19.62 | 15.01 | Supporting Information Figure S2C, Supporting Information Figure S2E |
|  |  | 0610 | Product | 20 | 37.09 | 51.05 | 3C, Supporting Information Figure S2C |
|  | Insoluble | 0610 | Substrate | 61 | 7.59 | 13.74 | 3D, Supporting Information Figure S2D |
|  |  |  |  | 48 | 17.79 | 24.59 |  |
|  |  |  |  | 41 | 23.20 | 25.39 |  |
|  |  | 0065 |  | 88 | 20.57 | 40.93 | Supporting Information Figure SD, Supporting Information Figure S3F |
| Bread | Insoluble | 0065 | Substrate | 51 | 12.81 | 8.50 | Supporting Information Figure SD, Supporting Information Figure S3F |
|  |  |  |  | 31 | 8.38 | 10.12 | Supporting Information Figure SD, Supporting Information Figure S3F |
